# Extremely low frequency wave localization via elastic foundation induced metamaterial with a spiral cavity

**DOI:** 10.1038/s41598-022-08002-9

**Published:** 2022-03-07

**Authors:** Myung Hwan Bae, Wonjae Choi, Jong Moon Ha, Miso Kim, Hong Min Seung

**Affiliations:** 1 Intelligent Wave Engineering Team, Korea Research Institute of Standards and Science (KRISS), Gajeong-ro 267, Yuseong-gu, Daejeon, 34113 South Korea; 2 Department of Precision Measurement, University of Science and Technology (UST), Gajeong-ro 267, Yuseong-gu, Daejeon, 34113 South Korea; 3 School of Advanced Materials Science and Engineering, Sungkyunkwan University (SKKU), Seobu-ro 2066, Jangan-gu, Suwon, 16419 South Korea

**Keywords:** Engineering, Physics

## Abstract

We proposed a metamaterial which exhibits elastic wave localization at extremely low frequencies. First, we opened an extremely low bandgap via elastic foundations. Subsequently, we investigated wave localization by imposing normal defect, which is widely used to capture waves in conventional wave localization systems. However, there were limitations: wave localization was not achieved when a weak bandgap is generated, and the operating frequency of localization is still in the upper part of the bandgap. To overcome wave localization via the normal defect, we proposed a novel metamaterial with a spiral cavity which can tune the resonating frequency depending on the length of the spiral path. By imposing on the spiral cavity inside the elastic foundation-induced metamaterial, we can shift the resonating frequency of the cavity down. Finally, we carried out wave simulations, not only to support the previous eigenfrequency study for the supercell, but also to verify that the finite-size metamaterial can also achieve wave localization at the extremely low frequencies. Through wave simulations, we could observe wave localization even at 77.3 Hz, which is definitely the lower part of the extremely low bandgap.

## Introduction

Over the last decade, focusing of elastic waves to one spot has attracted significant attention due to its promising potential in various practical applications, for example highly efficient energy harvesters, imaging systems with a high resolution, noise-cancelling systems, and vibration isolators. To achieve the spatial focusing of elastic waves, many researchers have fabricated periodic array of unit cells which are smaller than the wavelength, so called metamaterial. Since metamaterials can exhibit extraordinary phenomena, such as negative density, negative stiffness, or negative refraction, various ways have been presented to focus the elastic wave energy at a certain spot. Intuitively derived from classical wave devices, parabolic mirror metamaterials or funnel metamaterials have been proposed to gather the energy of wave at the focal point^1–3^. Furthermore, an elastic wave beyond a lens-like metamaterial can be localized at the focal point based on the gradient index of the phononic crystal^4–6^ or based on the metasurface^7^. In addition, graded-pillar shape metasurface^8^, acoustic black holes^9,10^, coiling up space^11^, and resonant defect inside a phononic crystal^12–14^ were presented as methods to localize the elastic wave.

Among the various metamaterials or metasurfaces used to focus elastic waves, resonant defect inside the phononic crystal is one of the most widely used since it is not only less affected by the direction of wave incidence, but also plane-type defect has an advantage in terms of the attachment of piezoelectric patch. Resonant defect inside phononic crystals can localize elastic wave with the generation of bandgap, the frequency range where wave propagation is forbidden. When the incident wave’s frequency is similar to the resonant frequency of the inside defect, the only inside defect shows standing wave motion since the neighboring unit cells form the bandgap. Based on this type of focusing mechanism by wave localization, recent studies on resonant defect inside phononic crystals have expanded to show a highly enhanced performance. Qi et al.^15^ theoretically showed that resonant defect inside acoustic metamaterial with piezoelectric transducers can produce electrical power from acoustic pressure. Park et al.^16^ experimentally verified wave localization based on an octagonal phononic crystal plate. To broaden the bandgap range, which is the sufficient condition of wave localization, Jo et al.^17^ designed a graded defect inside phononic crystal. In addition, to broaden the operating frequency of resonant defect, phononic crystals with double defect mode^18^ or phononic crystals with multi-mode cavity^19^ were proposed. Ma et al.^20^ presented a Helmholtz resonator-based metamaterial to induce a shift of the operating frequency. Jo et al.^21^ investigated the performance of wave localization depending on the location and the size of the defect. Moreover, Lee et al.^22^ achieved highly dense wave localization by optimizing the impedance between the phononic crystal and the piezoelectric sensors.

Despite recent explosive reports on wave localization, it has a limitation in the view of the frequency range. Few studies have been conducted on wave localization at low frequencies. This is because conventional phononic crystals have trouble generating the bandgap at the low frequency. Huge size of unit cell which corresponds to half of the wavelength should be designed to generate a Bragg-bandgap where wave motion is interfered by the reverse motion between neighboring unit cells^23–26^. Generally, locally resonance-based metamaterials can exhibit the low frequency bandgap by adjusting the ratio of the internal mass to the unit cell^27–30^; however, it is hard to design an inside defect that exhibits resonant motion within the bandgap range since locally resonant metamaterials have difficult achieving a broad bandgap. Several studies have opened the broad bandgap by inducing the conversion from translational motion to rotational motion with an extremely low rotational stiffness^31–33^. However, these bandgaps are not appropriate for achieving a system for wave localization due to its structural weakness caused by the very low rotational stiffness. Likewise, soft phononic crystal, which can exhibit low frequency bandgap, is not suitable for wave localization at the low frequencies considering that it is hard to assure structural reliability for a long time due to the large deformation^34–36^.

Here, it is worth noting that the extremely low bandgap can be induced by elastic foundation^37–41^. Wave localization is expected to be achieved at the extremely low frequency if we harness the bandgap induced by the elastic foundation, without the size problem of the Bragg-bandgap or without a narrow range of resonance bandgap. In addition, because the defect should be tuned to have a resonant frequency which corresponds to the extremely low bandgap, it is also worth noting studies on spiral metamaterials^42–49^. Shen et al.^42^ realized a spiral metamaterial plate that exhibits resonance motion at the low frequency. Additionally, highly tunable spiral metamaterials which include a magnetic force on the spiral unit cells were proposed to control the low frequency vibration^43,44^. Parametric modulations in spiral metamaterials enable manipulation of the direction of elastic wave propagation^45^ and present topological transitions^45,46^. Tian et al.^47^ proposed a metamaterial with an embedded spiral unit cell to merge the Bragg-bandgap and local resonance bandgap. Recently, the spiral metamaterials have been harnessed in various engineering applications such as breakwater systems against tidal waves^48^ and a flexible haptic sensor^49^.

**Figure 1:**
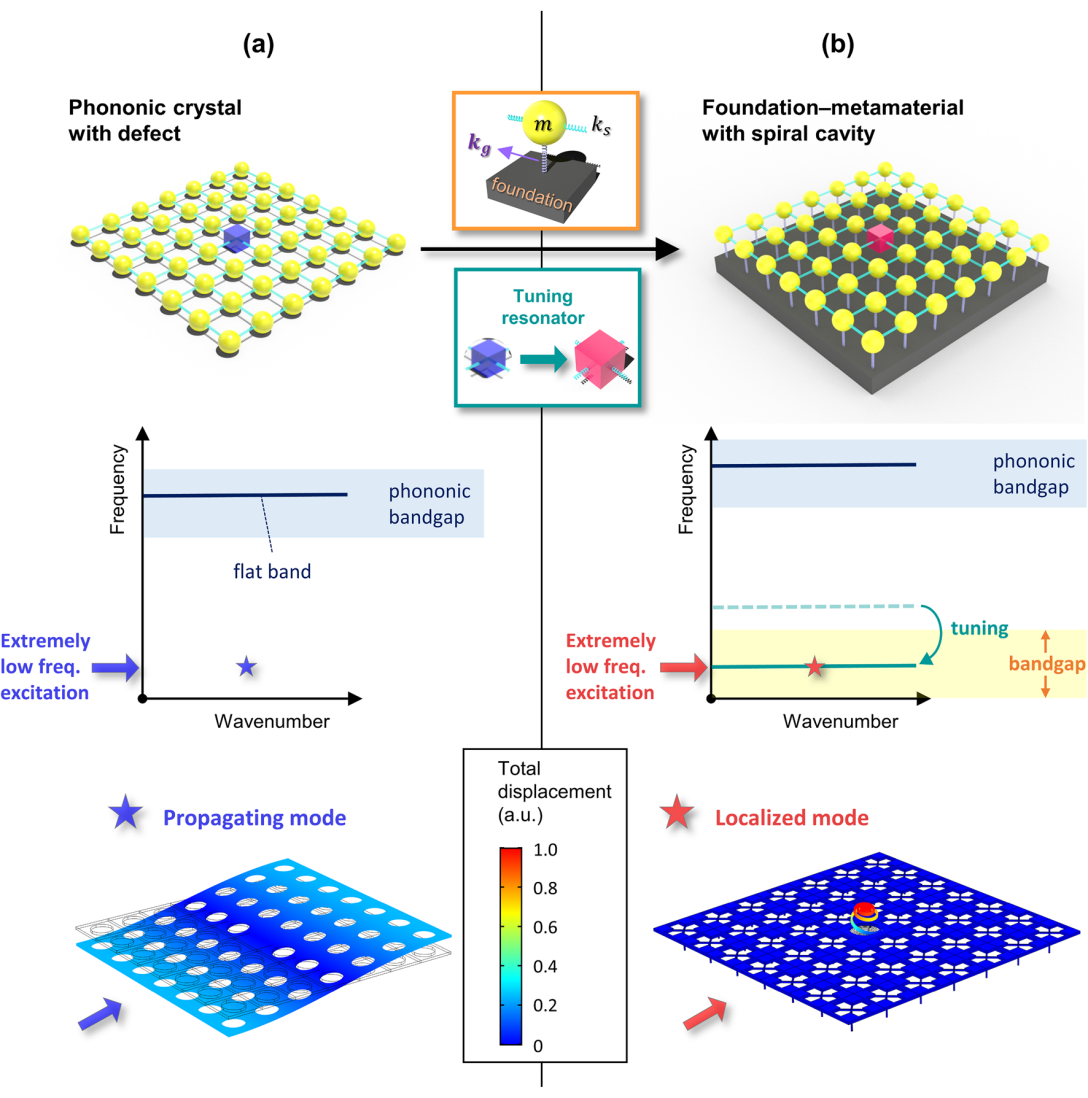
Scheme to achieve the extremely low frequency wave localization. (**a**) A general phononic crystal with defect and (**b**) Elastic foundation-induced metamaterial with a spiral cavity.

To achieve wave localization at the extremely low frequencies, in this paper, we propose a metamaterial by combining the elastic foundation-induced bandgap and a spiral cavity for tuning the resonant frequency. Figure 1 shows the scheme of the proposed metamaterial for the extremely low frequency localization (consisting of a discrete model, dispersion of the supercell, and wave mode at the extremely low frequencies). As in Fig. 1a general phononic crystal with a normal defect exhibits a high frequency bandgap and a flat band inside the bandgap. Since the bandgap is not generated at the extremely low frequencies, wave localization cannot be achieved via a general phononic crystal with normal defect. While, as shown in Fig. 1b, the proposed metamaterial exhibits the extremely low frequency bandgap by the elastic foundation and a flat band at the extremely low frequency due to the tunable resonating cavity depending on the spiral length. Therefore, wave localization at the extremely low frequency can be achieved.

## Results

### Elastic foundation induced an extremely low frequency bandgap

**Figure 2:**
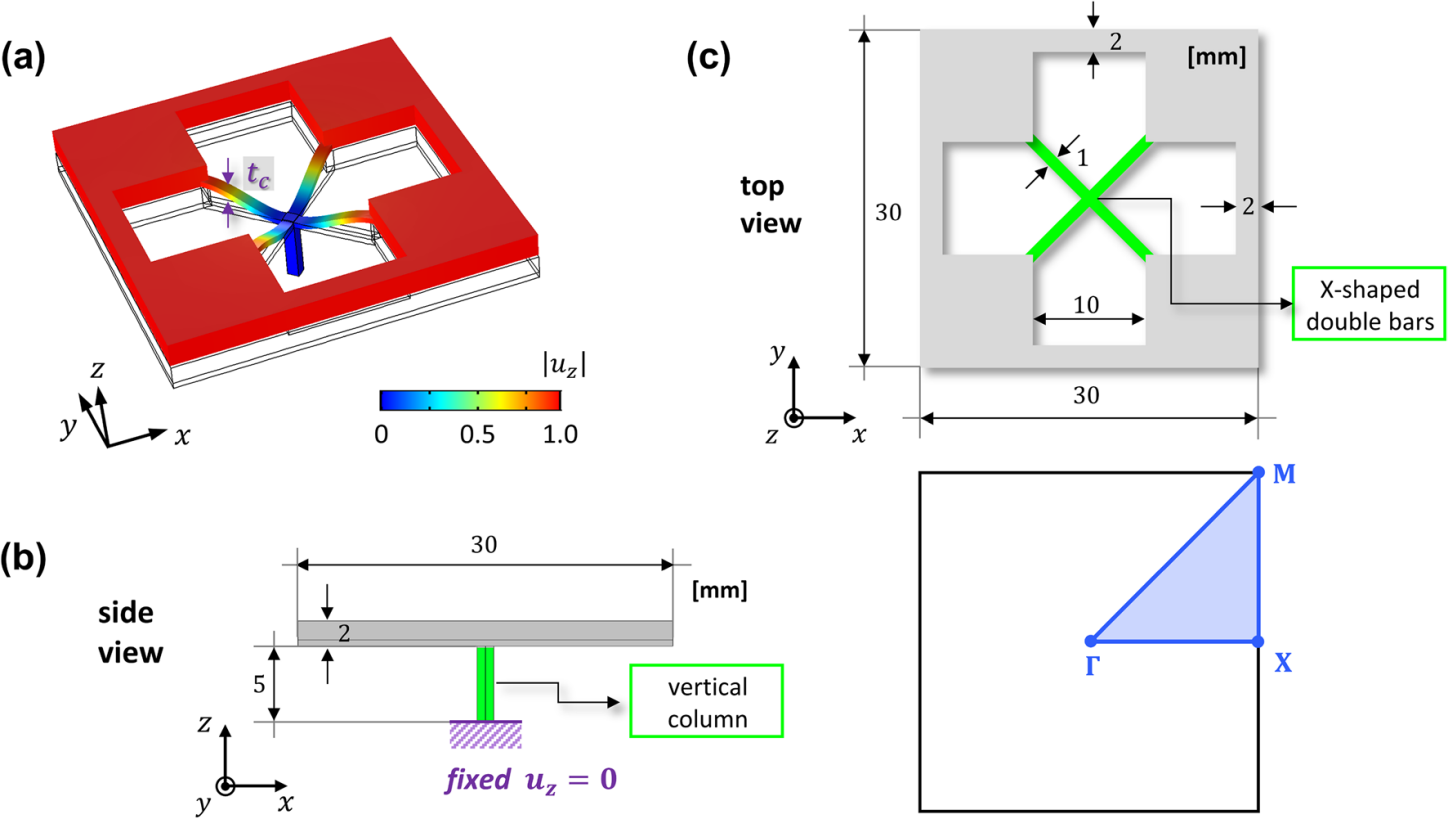
Unit cell with elastic foundation. (**a**) 3D view of the unit cell. (**b**) Geometric information from the side view. (**c**) Geometric information from the top view with the first irreducible Brillouin zone. (unit is in mm).

**Figure 3:**
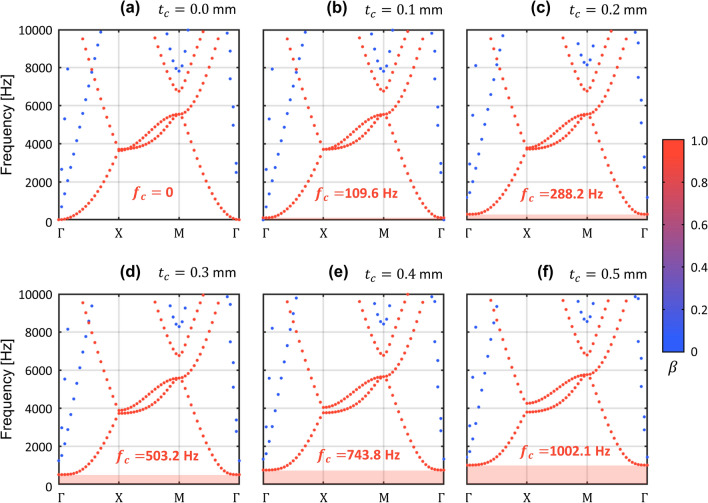
Band structure for varying stiffness of elastic foundation effect. The thickness of the X-shaped double bars $t_{c}$ is increased.

We introduce a metamaterial which can exhibit an extremely low frequency bandgap. Based on the existing study on the elastic foundation induced bandgap^37–41^, we designed the metamaterial by imposing an additional fixed boundary to each unit cell of a general phononic crystal plate, as shown in Fig. 1b. The specific design of the metamaterial for the extremely low frequency bandgap is presented in Fig. 2. The unit cell of our metamaterial is a 30 mm by 30 mm square plate with a thickness of 2 mm. Inside the square plate, a cross-shaped perforation is punched, and X-shaped double bars are designed across the perforation. In addition, at the centroid of the unit cell, 5 mm column whose bottom is fixed supports the X-shaped double bars. All materials of the geometric parts including the square plate, X-shaped double bars, and vertical column, are designed as aluminum, with Young’s modulus of 70 GPa, mass density of 2700 ${\text{kg/m}}^{3}$, and Poisson’s ratio of 0.33. In general, the dispersion relation in the irreducible Brillouin zone can be obtained by approaching the Bloch condition to the unit cell with the eigenvalue problem as:

1 $$ [{\text{K}} - \omega^{2} {\text{M}}]\phi = 0 $$

where ${\text{K}}$ and ${\text{M}}$ denote the stiffness matrix of the unit cell and the mass matrix of the unit cell, respectively, and $\phi$ represents the eigenmode of the unit cell for an angular frequency $\omega$. More details of the periodic solution from the unit cell study instead of solving the governing equation for an elastic wave that travels in an inhomogeneous elastic medium can be found in Supplementary S1. Since our metamaterial has an additional fixed boundary, which can be distinguished from the general phononic crystal plate, here we can simply formulate the equation of motion as new eigenvalue problem by considering an additional stiffness matrix from Eq. (1),

2 $$ [{\text{K + G}} - \omega^{2} {\text{M}}]\phi = 0 $$

where ${\text{G}}$ denotes the additional stiffness matrix by the elastic foundation effect. As in Fig. 2a, due to the X-shaped double bars and the vertical column, the unit cell exhibits motion by the elastic foundation. Note that the bottom of the vertical column is fixed at a zero displacement to express the elastic foundation, as shown in Fig. 2b. To show the band structure of the proposed metamaterial, we carry out the calculation to obtain $\omega$ with varying $\kappa$ as $\Gamma - {\rm X} - {\rm M} - \Gamma$ shown in Fig. 2c by using the structural module of COMSOL Multiphysics 5.4^50^. We performed a parametric study by increasing $t_{c}$ from 0 to 0.5 mm with an interval of 0.1 mm in order to intuitively show the tendency of bandgap generation at the extremely low frequency. One can easily figure out that parameter $t_{c}$ (highlighted as purple in Fig. 2a) is significant for determining the magnitude of matrix ${\text{G}}$ since the bending stiffness of the X-shaped double bars influences the z-directional displacement of the plate. It can also be interpreted that the greater the magnitude of parameter $t_{c}$, the more dominant the effect of the elastic foundation for the flexural wave motion. Figure 3 shows the dispersion relation depending on the thickness of the X-shaped double bars $t_{c}$ in the frequency range below 10,000 Hz. To focus on flexural modes, the polarization ratio $\beta$ is defined as^19^

3 $$ \beta = \frac{{\iiint_{v} {u_{z} u_{z}^{*} dxdydz}}}{{\iiint_{v} {(u_{x} u_{x}^{*} + u_{y} u_{y}^{*} + u_{z} u_{z}^{*} )dxdydz}}} $$

where the notation $*$ denotes the complex conjugate and $v$ represents the entire volumetric domain of the unit cell. If the polarization ratio $\beta$ is close to 1.0 (colored red), the flexural mode is dominant, whereas if $\beta$ is close to 0.0 (colored blue), the mode is an in-plane mode for the *z*-direction. As a result of a parametric study with an increase in $t_{c}$ from 0.0 to 0.5 mm, we can obtain an institution for bandgap generation at the extremely low frequency. Obviously, the bandgap cannot be observed in Fig. 3a where the case does not possess any elastic foundation. However, in Fig. 3b where $t_{c}$ is 0.1 mm, the extremely low frequency bandgap cut off at 109.6 Hz is generated. Even in the case where $t_{c}$ is 0.5 mm, the extremely low frequency complete bandgap is broadly generated from 0 to 1002.1 Hz, as shown in Fig. 3f. From this parametric study, it can be observed that the increase in the stiffness of the foundational springs leads to the generation of the broad complete bandgap covering the zero frequency. In addition, the extremely low frequency bandgap can be achieved through the elastic foundation. The effectiveness of harnessing the elastic foundation to obtain the extremely low frequency bandgap is compared with the bandgap by the general phononic crystal in Supplement S2.

### Wave localization using the normal defect

**Figure 4:**
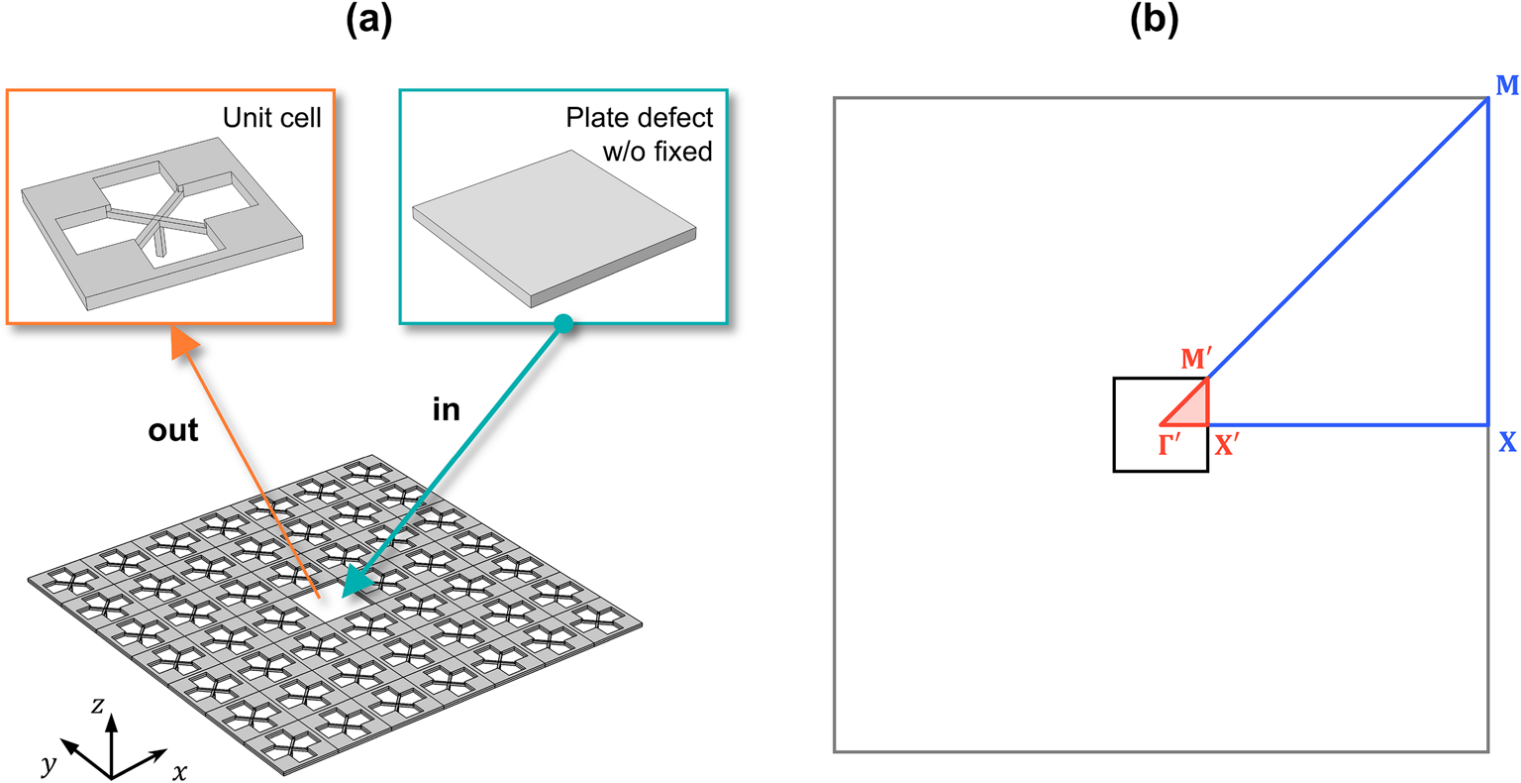
Wave localization using the normal defect. (**a**) Substituting the center unit cell with the plane-type defect. (**b**) The first irreducible Brillouin zone of the supercell.

**Figure 5:**
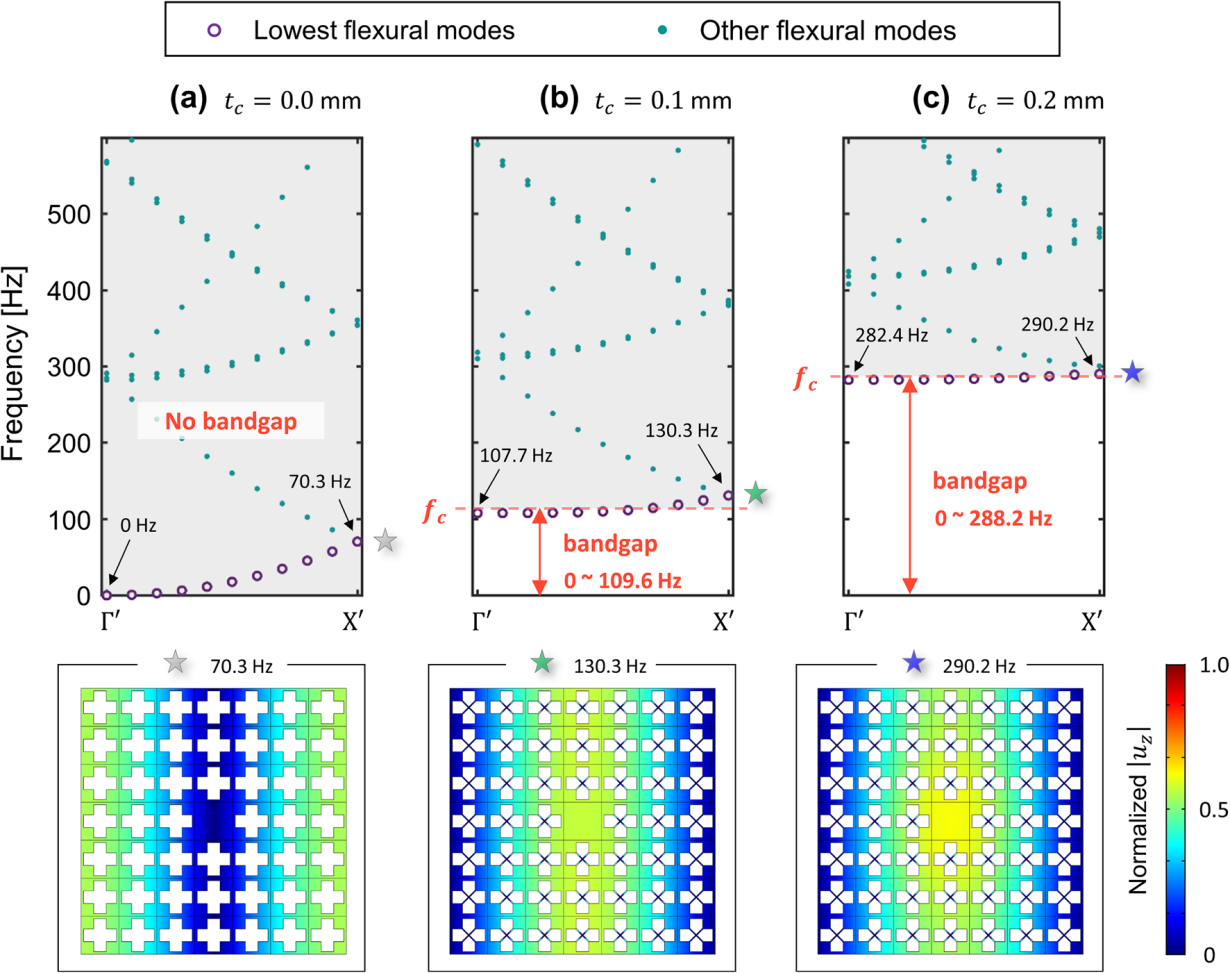
Not localized wave from the supercell analysis. $t_{c}$ is (**a**) 0.0 mm, (**b**) 0.1 mm and (**c**) 0.2 mm.

**Figure 6:**
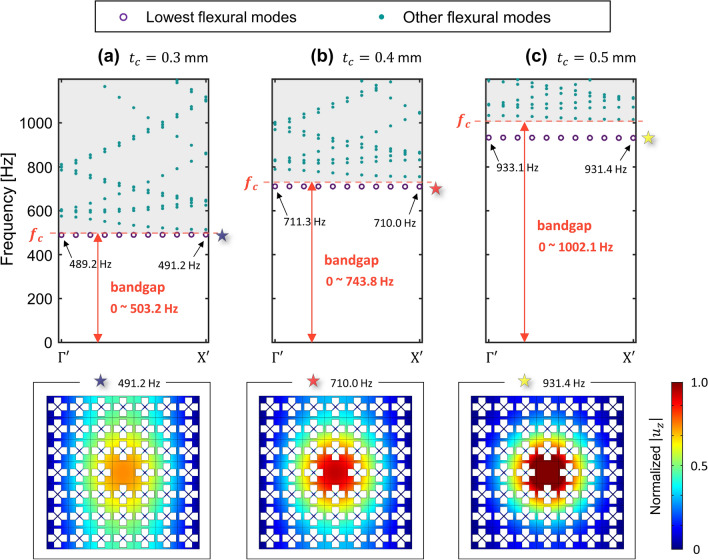
Localized wave from the supercell analysis. $t_{c}$ is (**a**) 0.3 mm, (**b**) 0.4 mm and (**c**) 0.5 mm.

Following the parametric study on the extremely low frequency bandgap, the wave localizations are achieved by imposing a plane-type defect unit cell inside the elastic foundation induced metamaterial. Since this plane-type defect without any perforation is widely adopted to achieve wave localization based on phononic crystal structures, it can be called as a normal defect. As presented in Fig. 4, we investigate wave localization using normal defect inside a 7 by 7 supercell. At the center of the 7 by 7 supercell, we substitute a unit cell into a normal defect without a fixed boundary, as in Fig. 4a. Additionally, the irreducible Brillouin zone is newly defined as $\Gamma ^{\prime} - {\rm X}^{\prime} - {\text{M}}^{\prime}$ to obtain the band structure of the normal defect inside the supercell, as shown in Fig. 4b. Along the $\Gamma ^{\prime} - {\rm X}^{\prime}$ boundary, we perform a parametric study of the supercell as the thickness of the X-shaped double bars $t_{c}$—increases from 0.0 to 0.5 mm with 0.1 mm intervals. From the supercell analysis, we plot the dispersion curve only for flexural modes whose polarization ratio $\beta$ is close to 1.0, as shown in Figs. 5 and 6. Figure 5 shows the cases where $t_{c}$ is 0.0 mm, 0.1 mm, and 0.2 mm, whereas Fig. 6 shows the cases where $t_{c}$ is 0.3 mm, 0.4 mm and 0.5 mm. Even at a glance, Figs. 5 and 6 clearly show a prominently different state of wave localization. The cases of Fig. 5 exhibit wave propagation rather than wave localization, whereas the cases of Fig. 6 exhibit wave localization at the defect unit cell. Figures 5 and 6 show the dispersion relation for the flexural wave by focusing on the lowest branch (marked as purple circle). On the dispersion curves of the flexural wave, the cut-off frequency or the range of the bandgap from the result of Fig. 3 are also expressed in orange color. The mode shapes below are extracted from the lowest flexural wave mode, where the wavevector $\kappa$ is equal to ${\rm X}^{\prime}$. To help compare the mode shapes, all mode shapes in Figs. 5 and 6 are normalized with the maximum displacement in the case of Fig. 6c where $t_{c}$ is 0.5 mm. First, because the extremely low frequency bandgap is not generated in the case of Fig. 5a, the propagating wave mode is observed at the point where the wavevector is ${\rm X}^{\prime }$ and the frequency is 70.3 Hz, as marked by the gray star. Although Fig. 5b, where $t_{c}$ is 0.1 mm, shows the extremely low frequency bandgap, the propagating wave mode is formed at the point where the wavevector is ${\rm X}^{\prime }$ and the frequency is 130.3 Hz, as marked by the green star. It can be interpreted that because 130.3 Hz is higher than the estimated cut-off frequency of 109.6 Hz, the wave cannot be captured at the defect although the bandgap is generated. Figure 5c, where $t_{c}$ is 0.2 mm also shows the propagating wave mode at the point marked with the blue star. Notwithstanding, the frequency of 290.2 Hz is slightly higher than the estimated cut-off frequency of 288.2 Hz, the wave mode at ${\rm X}^{\prime }$ does not match the bandgap and the wave cannot be captured at the defect.

However, in the cases shown in Fig. 6, we can observe the localized wave mode. In Fig. 6a, where $t_{c}$ is 0.3 mm, the frequency of the mode at ${\rm X}^{\prime }$, 491.2 Hz is lower than the estimated cut-off frequency of 503.2 Hz. Moreover, the slope of the lowest flexural branch is nearly zero. With the emergence of this flat branch, wave localization at the center defect can be achieved. Figure 6b, where $t_{c}$ is 0.4 mm, shows a more localized wave mode than the case of Fig. 6a. The frequency of the mode at ${\rm X}^{\prime }$, 710.0 Hz is sufficiently lower than the estimated cut-off frequency of 743.8 Hz, and even the slope of the lowest flexural branch has a slightly negative value. Similar to the shift from Fig. 6a, b, the shift from Fig. 6b, c also strongly supports wave localization phenomena. Figure 6c exhibits the most superior wave localization performance among the cases shown in Figs. 5 and 6. The frequency of the mode at ${\rm X}^{\prime }$, 931.4 Hz is lower than the cut-off frequency of 1002.1 Hz and the slope of the lowest branch has a slightly negative value. From this tendency, one can easily notice that the wave localization becomes stronger when the value of $t_{c}$ increases—as the cut off frequency becomes higher, the relative location of the flat band becomes lower. Through this study, we can achieve localization of the flexural wave in the extremely low bandgap under the condition where the lowest mode should exist inside the bandgap.

### Wave localization using spiral cavity

**Figure 7:**
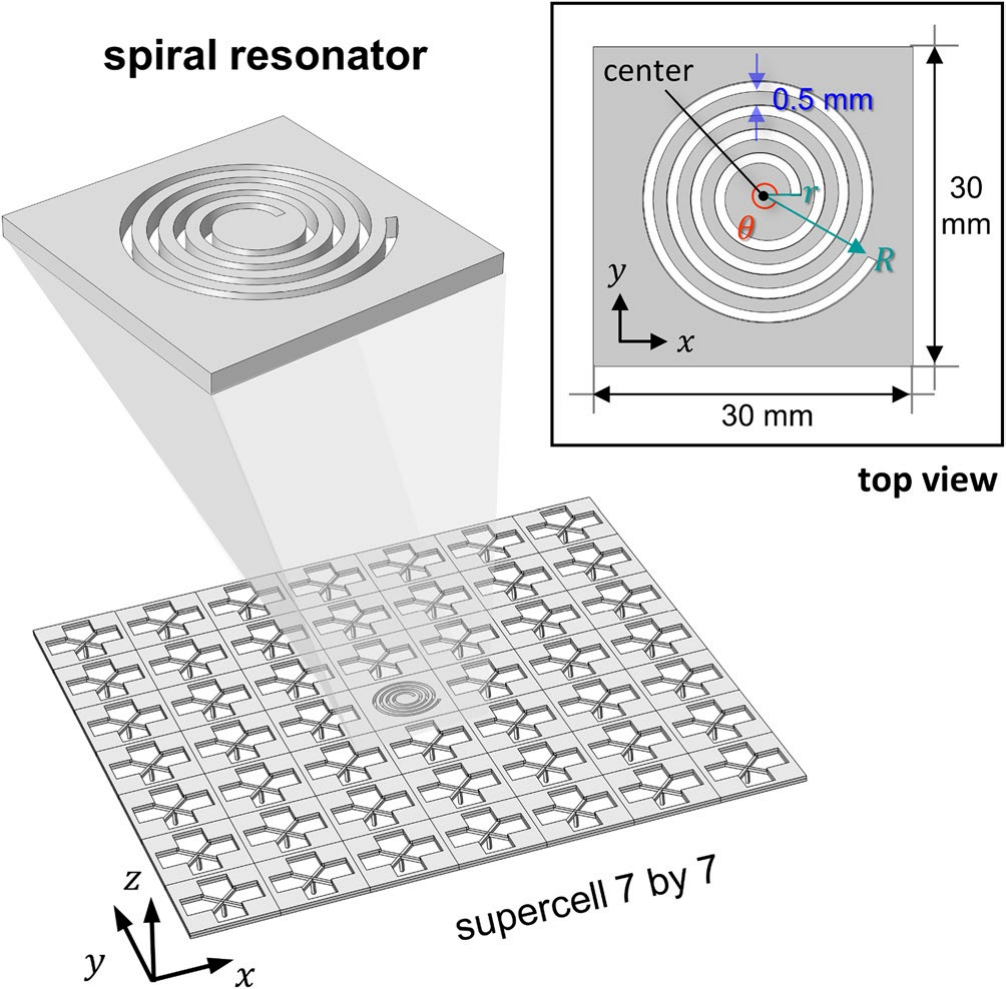
Substitution of the spiral cavity inside the elastic foundation-induced metamaterial. The inset shows the geometric information of the spiral cavity from the top view.

Although the proposed metamaterial with the elastic foundation can exhibit the extremely low frequency bandgap, wave localization cannot occur when the inside defect is not under the resonance state. Hence, to induce the extremely low frequency resonance of the inside defect, we propose a spiral cavity which can exhibit resonance modes at the extremely low frequencies instead of a normal defect. Recently, these spiral shape metamaterials have attracted much attention to achieve the low frequency bandgap based on the unit cell with a long spiral path^42–49^. Due to the unique structure of the spiral shape whose inner circle acts as a mass and whose rounding path acts as a bending spring, its effective density or stiffness can be tuned by focusing on the length of the spiral path or the ratio of the inner circle part. As shown in Fig. 7, we propose a novel metamaterial by combining the elastic foundation and the spiral cavity. The spiral path initiates at a point 9 mm apart in the *x*-direction from the centroid of the square lattice. The spiral path with a width of 0.5 mm can be defined by introducing an angle of the path $\theta$. Thus, the radius from the centroid to the end of the spiral path can be expressed as:

4 $$ {\text{R}} = 9 + \theta /\pi $$

since the gap between the spiral path is also 0.5 mm. According to Eq. (4), when the angle of the path $\theta$ is 1.00 $\pi$, R is 10 mm and when $\theta$ increases to 2.00 $\pi$, R becomes 11 mm.

**Figure 8:**
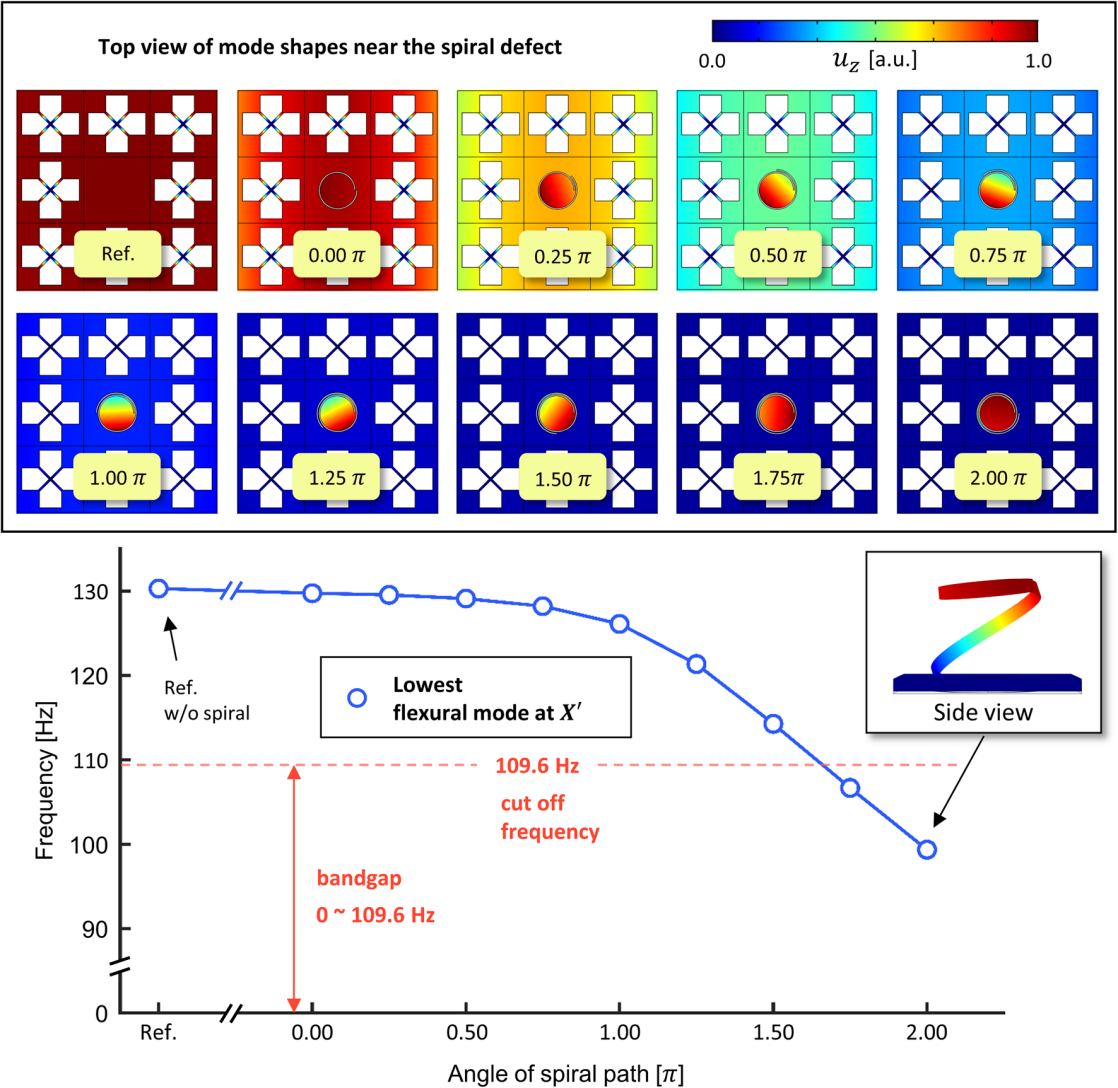
Frequency of the lowest flexural mode at ${\rm X}^{\prime}$ as the angle of the spiral path increases.

By increasing the length of the spiral path or the angle of the spiral path, we approached extremely low frequency wave localization for the case of Fig. 5b, where wave localization was not achieved. (In the case of Fig. 5b, where $t_{c}$ is 0.1 mm, the lowest flexural mode at ${\rm X}^{\prime }$ is formed at 130.3 Hz which is out of the bandgap whose cut off frequency is 109.6 Hz.) Fig. 8 shows the results of increasing the angle of the spiral path $\theta$ from 0.00$\pi$ to 2.00$\pi$ with an interval of 0.25$\pi$. In the case where $\theta$ is 0.00$\pi$, we obtained 129.7 Hz, which may have originated from the cut of the circular edge from the reference square lattice. The frequency of the lowest flexural mode at ${\rm X}^{\prime }$ continuously decreases as the angle of the spiral path varies up to 2.00$\pi$. When the angle of the spiral path is 2.00$\pi$, the lowest flexural mode at ${\rm X}^{\prime }$ is formed at 99.4 Hz. Note again that the cut-off frequency of the extremely low bandgap was 109.6 Hz under the condition where the thickness of the X-shaped double bars $t_{c}$ is 0.1 mm. Hence, one can interpret that the resonance frequency of the lowest flexural mode at ${\rm X}^{\prime}$ is shifted from out of the bandgap to the inside of the bandgap as the spiral cavity is tuned. Most importantly, wave localization is accomplished as shown in the subset of Fig. 8 which shows the top view of the mode shapes. Each mode shape is normalized with the maximum amplitude of each mode to easily grasp each localization state. Wave localization cannot be observed in the mode shapes of the reference case or the 0.00$\pi$ case. When introducing the spiral cavity with the path such as the cases for 0.25$\pi$ ~ 0.75$\pi$, a separation of motion between the cavity and the plate begins to occur. Eventually, the decrease in the frequency of the lowest flexural mode due to the increase in the angle of the spiral path induces wave localization, where the plate has nearly zero motion and only the cavity is vibrating. Through this investigation which introduces the spiral cavity, we showed wave localization at the extremely low frequency for the case where wave localization was not achieved by the conventional plane defect, normal defect.

**Figure 9:**
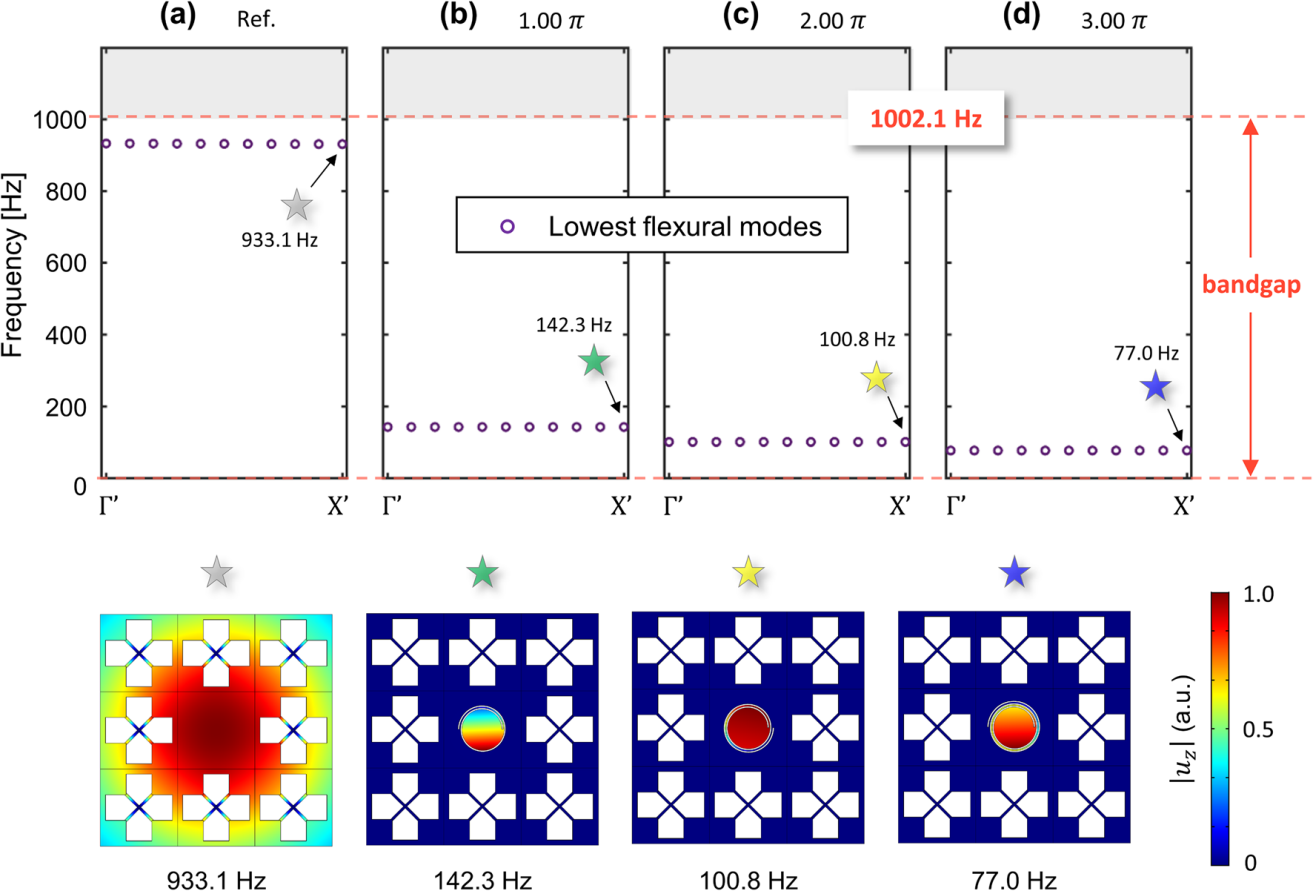
Band-structure by the supercell analysis for elastic foundation metamaterials with spiral cavities when $t_{c}$ is 0.5 mm. (**a**) Reference cavity without the spiral path. (**b**) The angle of the spiral path is 1$\pi$, (**c**) the angle of the spiral path is 2$\pi$, and (**d**) the angle of the spiral path is 3$\pi$.

Next, we also show the tuning the cavity mode when the resonance frequency of the cavity still exists in the high part of the bandgap. In Fig. 6c where $t_{c}$ is 0.5 mm, wave localization was achieved at a frequency of 931.4 Hz which is lower than the cut-off frequency of the bandgap of 1002.1 Hz. We consider three cases with spiral path angles of 1.00$\pi$, 2.00$\pi$, and 3.00$\pi$. Figure 9 shows the result with the reference case for the normal defect. To highlight the shift of the wave localization mode, we plot only the lowest flexural modes. Moreover, the plots of the mode shapes neighboring the cavity cell correspond to each mode at ${\rm X}^{\prime }$, marked by the colored stars. Each mode shape is normalized with each maximum *z*-directional amplitude $u_{z}$. First, as shown in Fig. 9a, the flat band is located at 933.1 Hz in the upper part of bandgap. However, Fig. 9b shows that when the angle of the spiral path is 1.00$\pi$, the location of the flat band is significantly lower (to 142.3 Hz where the lower part of bandgap and the mode shape are marked with a green star). Also, Fig. 9c, when the angle of the spiral path is 2.00$\pi$, shows a flat band at 100.8 Hz and Fig. 9d, when the angle of the spiral path is 3.00$\pi$, shows a flat band which exists at 77.0 Hz. In each case, flat bands definitely exist in the lower part of the bandgap whose range is from 0 Hz to 1002.1 Hz. The localized mode can shift from the upper part of the bandgap to the lower part of bandgap by introducing the spiral cavity which is tunable depending on the spiral path.

### Verification

**Figure 10:**
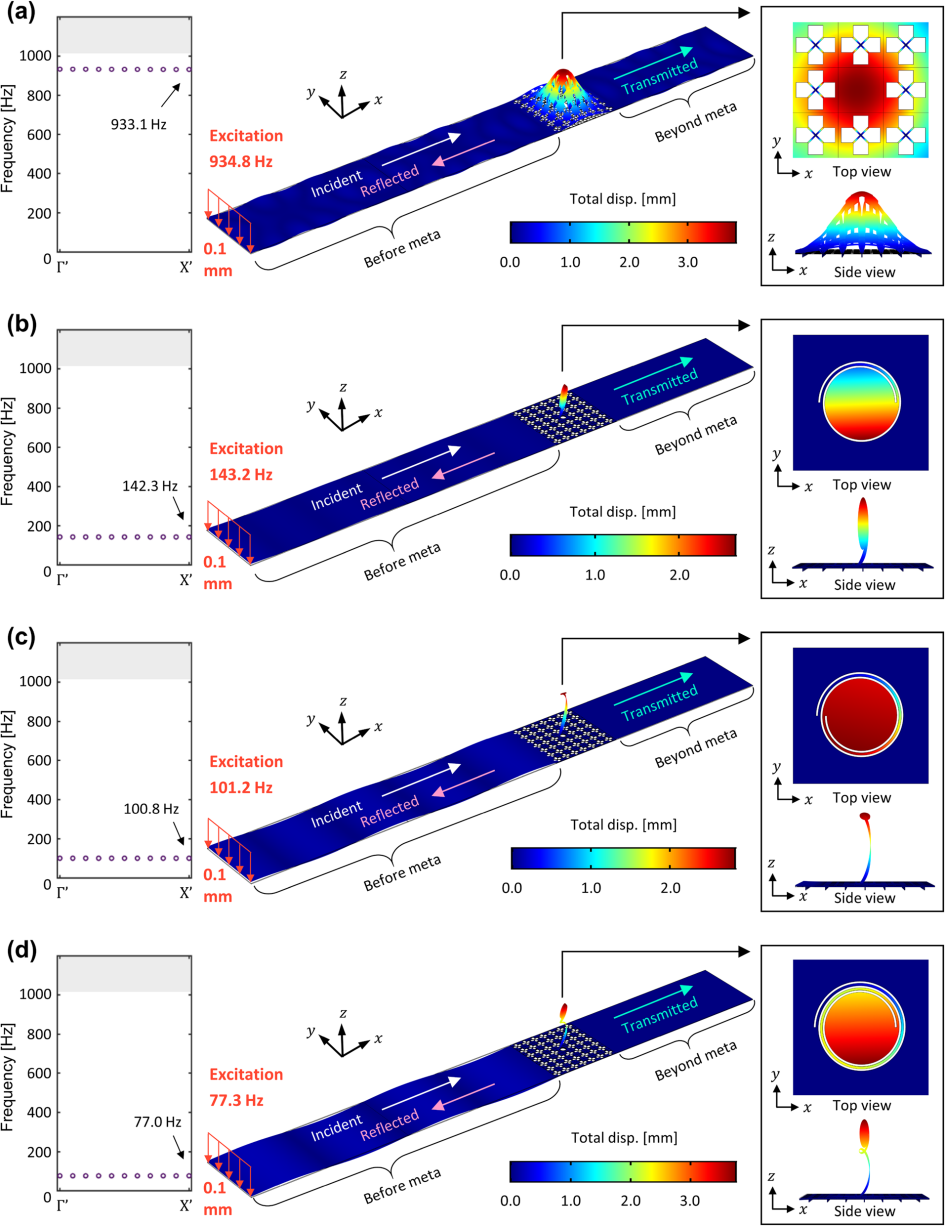
Simulation results. (**a**) Normal defect and (**b**, **c**, **d**) path of spiral cavity: 1$\pi$, 2$\pi$, and 3$\pi$.

Since the analysis of the previous sections has a limitation due to assumption of the infinite periodicity, we carried out wave propagation simulation to verify the wave localization at the extremely low frequencies for the proposed elastic foundation-induced metamaterial with the spiral cavity. The flexural wave localization is verified based on the cases of the previous section, such as 142.3 Hz, 100.8 Hz, and 77.0 Hz. The detailed setup of the wave simulation can be found in the Method section. Figure 10 shows the wave simulation result when the lowest flexural waves are localized. In each case, a harmonic displacement of 0.1 mm is imposed on the line source and captured the mode shapes when wave localization is achieved. The insets show the top and side views of the cavity part. It can be seen that the flexural wave from the line source cannot propagate beyond the metamaterial since wave localization is achieved in each case. As shown in Fig. 10a, the lowest flexural wave is localized at the frequency of 934.8 Hz with the maximum amplitude of 3.83 mm. As shown in Fig. 10b, the lowest flexural wave is localized at a frequency of 143.2 Hz with a maximum amplitude of 2.69 mm in the case where the angle of the spiral path is 1.00$\pi$. Furthermore, the lowest flexural wave is localized at 101.2 Hz with a maximum amplitude of 2.84 mm in Fig. 10c where the angle of the spiral path is 2.00$\pi$. Here, when the direction of wave incidence and the shape of the spiral path are considered, the result is obtained for the spiral cavity which was rotated by 180$^\circ$. Finally, as shown in Fig. 10d, in the case where the angle of the spiral path is 3.00$\pi$, the lowest flexural wave is localized at a frequency of 77.3 Hz with a maximum amplitude of 3.76 mm. The gaps in the operating frequency of the cavity between the supercell analysis and wave simulation are insignificant—even in the case of Fig. 10d where the angle of the spiral path is 3.00$\pi$, the gap is only 0.3 Hz. Although the maximum amplitudes used in the normalization of each mode shape are measured with different values from 3.83, 2.69, 2.84, and 3.76 mm, the comparison of localization performance between the four cases is not valid since we sweep the frequencies with an interval of 0.1 Hz. A finer frequency interval should be applied to compare the wave localization performances between the four cases. However, since the main goal of this study is not to enhance the performance of wave localization but to show the extremely low frequency wave localization, we conclude that wave localization in the extremely low frequencies is achieved with a similar level of performance, given that the scale of the maximum amplitude for each case is similar. From the results of the wave simulations, we verified that the proposed elastic foundation-induced metamaterial with the spiral cavity can achieve wave localization at the extremely low frequencies, especially for the metamaterial with finite size.

## Methods

**Figure 11:**
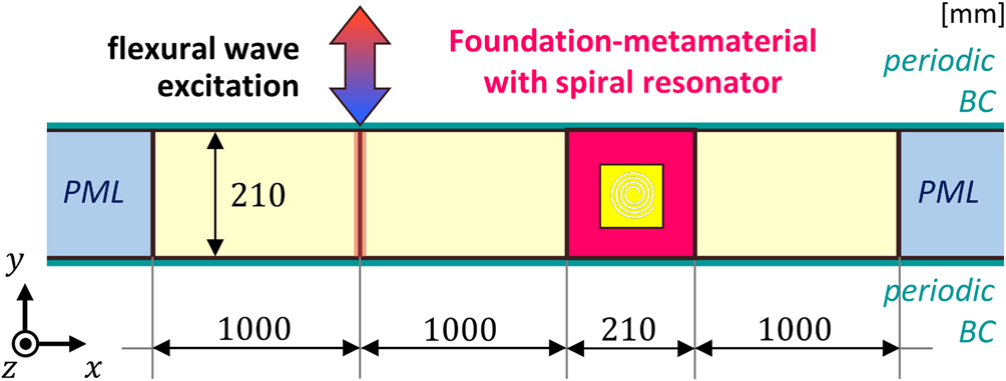
Setup for wave simulations in the frequency domain.

The setup of the wave simulation is shown in Fig. 11. First, we numerically design a 210 mm by 210 mm metamaterial with 48 unit cells with the elastic foundations and one spiral cavity to create the finite version of the 7 by 7 supercell. Next, we add aluminum layers with a width of 2000 mm, height of 210 mm, and a thickness of 2 mm at the left side of the metamaterial, with half of this layer added to the right side of the metamaterial. Additionally, to prevent the wave from reflecting, we set the perfect matched layer at the end of both the left and right sides and define the periodic boundary at the upper and the lower parts. We mark a line 1000 mm from the metamaterial to impose a harmonic displacement of 0.1 mm. Wave simulations are carried out based on the frequency domain module in COMSOL Multiphysics. Since the frequency of wave localization from the supercell analysis based on eigenfrequency study differs slightly from the wave simulation based on the frequency domain study, we imposed a sweep of the frequencies on the line source to draw the localized mode.

## Discussion

We have proposed a metamaterial which exhibits wave localization at the extremely low frequency. First, by adding elastic foundations to each unit cell, we opened the extremely low bandgap. Subsequently, we investigated wave localization by imposing the normal defect, which is widely used to capture waves in the conventional wave localization systems. However, there were two limitations: wave localization was not achieved when a weak bandgap was generated, and the operating frequency of localization was still in the upper part of the bandgap. To overcome wave localization via normal defect, we proposed a novel metamaterial with a spiral cavity which can tune the resonating frequency depending on the length of the spiral path. By imposing on the spiral cavity inside the elastic foundation-induced metamaterial, we can shift down the resonating frequency of the cavity. We showed how to achieve wave localization for the cases in which wave localization was not achieved via normal defect by shifting down the operating frequency to 99.4 Hz. In addition, for the case where $t_{c}$ is 0.5 mm, we achieved a shift of operating frequency from 933.1 to 77.0 Hz with an increase in the spiral path. Finally, wave simulations were carried out not only to support the previous eigenfrequency study for the supercell, but also to verify that the finite-size metamaterial can also achieve wave localization at the extremely low frequencies. Even in the wave simulations, we observed wave localization at 77.3 Hz, which is definitely the lower part of extremely low bandgap.

The feasibility of wave localization at the extremely low frequency is not entirely certain because our study is based on numerical simulations, including eigenfrequency study for the supercells and wave propagation in the frequency domain. To obtain the more complete feasibility, a fixed boundary to induce zero motion on the unit cells and consideration of the deflection of the X-shaped bar by the gravity effect would be required. Although providing a quasi-zero motion can induce a very low frequency bandgap and increasing $t_{c}$ can bring a the more suitable wave localization condition due to the higher cut off frequency, it has a slightly different condition from the cases covered in this study. We hope that frontier research on the realization of the exquisite metamaterials can overcome this limitation with the analysis of quantitative approaches under external conditions, such as zero motion and gravity.

Throughout this study, it is possible to achieve wave localization at the extremely low frequency with the compact size of the metamaterial, a combination of the bandgap by elastic foundation effect and the spiral cavity with the long spiral path. Our study at the extremely low frequency can expand the scope of new research on wave localization, which has mostly focused on the high frequency. In addition, our findings are expected to have high engineering potential for various practical applications based on wave manipulation or vibration shielding by achieving wave localization at the extremely low frequencies where waves are difficult to dissipate in nature. (i.e., a highly sensitive sensing system to detect the impalpable oscillation can be achieved by amplifying the waves, a highly efficient dissipation system by simply adding dissipative material just to the spiral cavity, and an extremely dense mechanical-energy harvesting system by attaching the piezoelectric material on the spiral cavity.)

## Supplementary Information

Supplementary Information 1.

Supplementary Information 2.

## References

[1] Carrara M, Cacan MR, Leamy MJ, Ruzzene M, Erturk A (2012). Dramatic enhancement of structure-borne wave energy harvesting using an elliptical acoustic mirror. Appl. Phys. Lett..

[2] Tol S, Degertekin FL, Erturk A (2017). Structurally embedded reflectors and mirrors for elastic wave focusing and energy harvesting. J. Appl. Phys..

[3] Carrara M, Cacan MR, Toussaint J, Leamy MJ, Ruzzene M, Erturk A (2013). Metamaterial-inspired structures and concepts for elastoacoustic wave energy harvesting. Smart Mater. Struct..

[4] Tol S, Degertekin FL, Erturk A (2016). Gradient-index phononic crystal lens-based enhancement of elastic wave energy harvesting. Appl. Phys. Lett..

[5] Hyun J, Choi W, Kim M (2019). Gradient-index phononic crystals for highly dense flexural energy harvesting. Appl. Phys. Lett..

[6] Tol S, Degertekin FL, Erturk A (2017). Phononic crystal Luneburg lens for omnidirectional elastic wave focusing and energy harvesting. Appl. Phys. Lett..

[7] Qi S, Assouar B (2017). Acoustic energy harvesting based on multilateral metasurfaces. Appl. Phys. Lett..

[8] De Ponti JM, Colombi A, Ardito R, Braghin F, Corigliano A, Craster RV (2020). Graded elastic metasurface for enhanced energy harvesting. New J. Phys..

[9] Ji H, Liang Y, Qiu J, Cheng L, Wu Y (2019). Enhancement of vibration based energy harvesting using compound acoustic black holes. Mech. Syst. Signal Process..

[10] Zhao L, Conlon SC, Semperlotti F (2014). Broadband energy harvesting using acoustic black hole structural tailoring. Smart Mater. Struct..

[11] Sun KH, Kim JE, Kim J, Song K (2017). Sound energy harvesting using a doubly coiled-up acoustic metamaterial cavity. Smart Mater. Struct..

[12] Wu LY, Chen LW, Liu CM (2009). Acoustic energy harvesting using resonant cavity of a sonic crystal. Appl. Phys. Lett..

[13] Wang WC, Wu LY, Chen LW, Liu CM (2010). Acoustic energy harvesting by piezoelectric curved beams in the cavity of a sonic crystal. Smart Mater. Struct..

[14] Miniaci M, Gliozzi AS, Morvan B, Krushynska A, Bosia F, Scalerandi M, Pugno NM (2017). Proof of concept for an ultrasensitive technique to detect and localize sources of elastic nonlinearity using phononic crystals. Phys. Rev. Lett..

[15] Qi S, Oudich M, Li Y, Assouar B (2016). Acoustic energy harvesting based on a planar acoustic metamaterial. Appl. Phys. Lett..

[16] Park CS, Shin YC, Jo SH, Yoon H, Choi W, Youn BD, Kim M (2019). Two-dimensional octagonal phononic crystals for highly dense piezoelectric energy harvesting. Nano Energy.

[17] Jo SH, Yoon H, Shin YC, Youn BD (2020). A graded phononic crystal with decoupled double defects for broadband energy localization. Int. J. Mech. Sci..

[18] Jo SH, Yoon H, Shin YC, Kim M, Youn BD (2020). Elastic wave localization and harvesting using double defect modes of a phononic crystal. J. Appl. Phys..

[19] Ma TX, Fan QS, Li ZY, Zhang C, Wang YS (2020). Flexural wave energy harvesting by multi-mode elastic metamaterial cavities. Extreme Mech. Lett..

[20] Ma K, Tan T, Yan Z, Liu F, Liao WH, Zhang W (2021). Metamaterial and Helmholtz coupled resonator for high-density acoustic energy harvesting. Nano Energy.

[21] Jo SH, Yoon H, Shin YC, Choi W, Park CS, Kim M, Youn BD (2020). Designing a phononic crystal with a defect for energy localization and harvesting: Supercell size and defect location. Int. J. Mech. Sci..

[22] Lee TG, Jo SH, Seung HM, Kim SW, Kim EJ, Youn BD, Kim M (2020). Enhanced energy transfer and conversion for high performance phononic crystal-assisted elastic wave energy harvesting. Nano Energy.

[23] Kushwaha MS, Halevi P, Dobrzynski L, Djafari-Rouhani B (1993). Acoustic band structure of periodic elastic composites. Phys. Rev. Lett..

[24] Sigalas M, Economou EN (1993). Band structure of elastic waves in two dimensional systems. Solid State Commun..

[25] Bae MH, Oh JH (2020). Amplitude-induced bandgap: New type of bandgap for nonlinear elastic metamaterials. J. Mech. Phys. Solids.

[26] Pelat A, Gallot T, Gautier F (2019). On the control of the first Bragg band gap in periodic continuously corrugated beam for flexural vibration. J. Sound Vib..

[27] Liu Z, Zhang X, Mao Y, Zhu YY, Yang Z, Chan CT, Sheng P (2000). Locally resonant sonic materials. Science.

[28] Fang N, Xi D, Xu J, Ambati M, Srituravanich W, Sun C, Zhang X (2006). Ultrasonic metamaterials with negative modulus. Nat. Mater..

[29] Zhu R, Liu XN, Hu GK, Sun CT, Huang GL (2014). A chiral elastic metamaterial beam for broadband vibration suppression. J. Sound Vib..

[30] Jung J, Goo S, Wang S (2020). Investigation of flexural wave band gaps in a locally resonant metamaterial with plate-like resonators. Wave Motion.

[31] Oh JH, Choi SJ, Lee JK, Kim YY (2018). Zero-frequency Bragg gap by spin-harnessed metamaterial. New J. Phys..

[32] Park S, Jeon W (2021). Ultra-wide low-frequency band gap in a tapered phononic beam. J. Sound Vib..

[33] Park HW, Seung HM, Kim M, Choi W, Oh JH (2021). Continuum flexural metamaterial for broadband low-frequency band gap. Phys. Rev. Appl..

[34] Bayat A, Gordaninejad F (2015). Band-gap of a soft magnetorheological phononic crystal. J. Vib. Acoust..

[35] Wu B, Zhou W, Bao R, Chen W (2018). Tuning elastic waves in soft phononic crystal cylinders via large deformation and electromechanical coupling. J. Appl. Mech..

[36] Nguyen BH, Zhuang X, Park HS, Rabczuk T (2019). Tunable topological bandgaps and frequencies in a pre-stressed soft phononic crystal. J. Appl. Phys..

[37] Yao S, Zhou X, Hu G (2010). Investigation of the negative-mass behaviors occurring below a cut-off frequency. New J. Phys..

[38] Yu D, Wen J, Shen H, Xiao Y, Wen X (2012). Propagation of flexural wave in periodic beam on elastic foundations. Phys. Lett. A.

[39] Liu Y, Shen X, Su X, Sun CT (2016). Elastic metamaterials with low-frequency passbands based on lattice system with on-site potential. J. Vib. Acoust..

[40] Chen Z, Wang G, Zhou W, Lim CW (2021). Elastic foundation induced wide bandgaps for actively-tuned topologically protected wave propagation in phononic crystal beams. Int. J. Mech. Sci..

[41] Han L, Zhang Y, Li XM, Jiang LH, Chen D (2015). Flexural vibration reduction of hinged periodic beam–foundation systems. Soil Dyn. Earthq. Eng..

[42] Shen L, Wu JH, Zhang S, Liu Z, Li J (2015). Low-frequency vibration energy harvesting using a locally resonant phononic crystal plate with spiral beams. Mod. Phys. Lett. B.

[43] Bilal OR, Foehr A, Daraio C (2017). Bistable metamaterial for switching and cascading elastic vibrations. Proc. Natl. Acad. Sci..

[44] Jiang T, He Q (2017). Dual-directionally tunable metamaterial for low-frequency vibration isolation. Appl. Phys. Lett..

[45] Foehr A, Bilal OR, Huber SD, Daraio C (2018). Spiral-based phononic plates: From wave beaming to topological insulators. Phys. Rev. Lett..

[46] Li S, Yang J (2021). Topological transition in spiral elastic valley metamaterials. Phys. Rev. Appl..

[47] Tian X, Chen W, Gao R, Liu S (2021). Merging bragg and local resonance bandgaps in perforated elastic metamaterials with embedded spiral holes. J. Sound Vib..

[48] Ruan Y, Liang X, Hua X, Zhang C, Xia H, Li C (2021). Isolating low-frequency vibration from power systems on a ship using spiral phononic crystals. Ocean Eng..

[49] Bilal OR, Costanza V, Israr A, Palermo A, Celli P, Lau F, Daraio C (2020). A flexible spiraling-metasurface as a versatile haptic interface. Advanced Materials Technologies.

[50] COMSOL Multiphysics® v. 5.4. www.comsol.com. COMSOL AB, Stockholm, Sweden.

