# Supplementary figures and images for: Extremely low frequency wave localization via elastic foundation induced metamaterial with a spiral cavity
Source: Sci Rep. 2022 Mar 7;12:3993. doi: 10.1038/s41598-022-08002-9 (PMC8901657; doi:10.1038/s41598-022-08002-9)

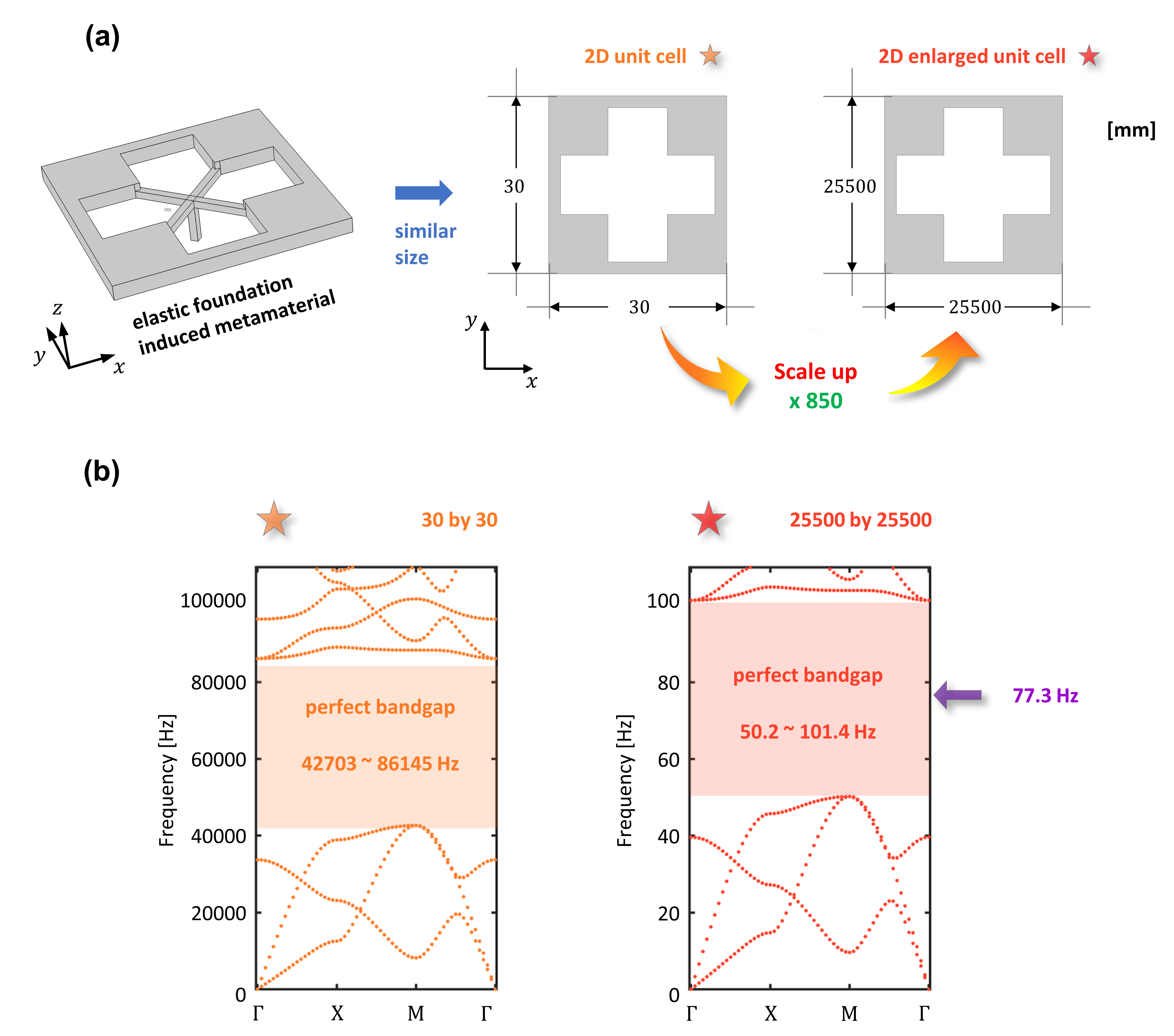

Supplement: Supplementary file 1 — Supplementary Information 1. [file 41598_2022_8002_MOESM1_ESM.tif]
