# Supplementary material for: Extremely low frequency wave localization via elastic foundation induced metamaterial with a spiral cavity
Source: Sci Rep. 2022 Mar 7;12:3993. doi: 10.1038/s41598-022-08002-9 (PMC8901657; doi:10.1038/s41598-022-08002-9)
Supplement: Supplementary file 2 — Supplementary Information 2. [file 41598_2022_8002_MOESM2_ESM.docx]

**Extremely Low Frequency Wave Localization via Elastic Foundation Induced Metamaterial with a Spiral Cavity**

**Myung Hwan Bae^a^, Wonjae Choi^a^, Jong Moon Ha^a^, Miso Kim^b^ and Hong Min Seung^a^*^*^***

*^a^ AI Metamaterial Research Team, Korea Research Institute of Standards and Science (KRISS), Gajeong-ro 267, Yuseong-gu, Daejeon 34113, South Korea*

*^b^ School of Advanced Materials Science and Engineering, Sungkyunkwan University (SKKU),*

*Seobu-ro 2066, Jangan-gu, Suwon 16419, South Korea*

*^*^ Corresponding Author, Email: shm@kriss.re.kr, Tel: +82 42 868 5664*

**Supplement 1.**

For the general phononic crystal plate, as shown in Fig.1-(a), the governing equation for an elastic wave that travels in an inhomogeneous elastic medium is given as follows:

$\nabla\cdot\left(C(\overrightarrow{r}):\nabla u(\overrightarrow{r})\right)=\rho(\overrightarrow{r})\frac{\partial^{2}u(\overrightarrow{r})}{\partial t^{2}}$ (S1)

where is the gradient operator, $\overrightarrow{r}=(x,y,z)$ is the position vector, and $C(\overrightarrow{r})$, $u(\overrightarrow{r})$, and $\rho(\overrightarrow{r})$ are the elastic tensor, displacement vector, and density tensor, respectively. The solution of Eq. (S1), $u(\overrightarrow{r},t)$ can be expressed from the dispersion relation between the angular frequency $\omega$, and the wavevector $\vec{\kappa}$,

$u(\overrightarrow{r},t)=\widehat{u}(\overrightarrow{\kappa}(\omega))\exp[(i(\omega t-\overrightarrow{\kappa}\overrightarrow{r}))]$ (S2)

where $\widehat{u}(\overrightarrow{\kappa}(\omega))$ is an amplitude modulation function that depends on the dispersion relation between $\omega$ and $\vec{\kappa}$. Noting that the elastic medium is assumed to be periodic media in the *x*-direction and the *y*-direction as shown in Fig.1-(a), one can define the wavevector $\vec{\kappa}$ as $(\kappa_{x},\kappa_{y})$ by using the following lattice vector,

$\kappa_{x}=\frac{2\pi(e_{y}\times e_{z})}{e_{x}\cdot(e_{y}\times e_{z})}; \kappa_{y}=\frac{2\pi(e_{x}\times e_{z})}{e_{y}\cdot(e_{x}\times e_{z})}$ (S3)

where $\overrightarrow{e_{x}}=(\overrightarrow{a_{x}},0,0)$,$\overrightarrow{e_{y}}=(0,\overrightarrow{a_{y}},0)$, and$\overrightarrow{e_{z}}=(0,0,1)$. Since we consider isotropic periodic media, the periodic length vectors between the unit cells in the *x*-direction $\overrightarrow{a_{x}}$ and the *y*-direction $\overrightarrow{a_{y}}$ are the same as the constant $a$. Then, the solution for Eq. (S1) can be written with the periodic function by Bloch theorem,

$u(\overrightarrow{r}+a,t)=u(\overrightarrow{r},t)\exp(i\overrightarrow{\kappa}\cdot a)$ (S4)

That is, solving the elastic wave equation for the phononic crystal plate can be substituted to solve the solution for the unit cell with the periodic boundary, as shown in Eq. (S4). By approaching the Bloch condition to the unit cell with an interval of $a$, we can obtain the dispersion relation in the irreducible Brillouin zone. In the unit cell, Eq. (S1) can be equal to the eigenvalue problem as:

(S5)

where $\text{K}$ and $\text{M}$ denote the stiffness matrix of the unit cell and the mass matrix of the unit cell, respectively, and represents the eigenmode of the unit cell for an angular frequency of $\omega$. Returning to the proposed metamaterial (the phononic crystal plate with the elastic foundation) with an additional fixed boundary, which can be distinguished from the general phononic crystal plate, we can formulate the equation of motion as the eigenvalue problem by simply adding $\text{G}$ in the matrix from Eq. (S5),

(S6)

where $\text{G}$ denotes the stiffness matrix by the effect of the springs linking each unit cell to the fixed-elastic foundation.

**Supplement 2.**

Here, to demonstrate the superiority of the elastic foundation-induced metamaterial, a size comparison between our metamaterial and general phononic crystal is presented when they form the extremely low frequency bandgap. In the manuscript, except for the elastic foundation, the unit cell of the metamaterial is a thin plate with a width of 30 mm, a height of 30 mm and a thickness of 2 mm. For convenience, we show the bandgap of the 2D unit cell of a general phononic crystal whose size is similar to that of our metamaterial. As shown in Fig.S1-(a), a small phononic crystal is based on our metamaterial and a large phononic crystal is obtained by enlarging the small phononic crystal 850 times. Fig.S1-(b) shows the bandgap of each phononic crystal. The small phononic crystal exhibits a bandgap ranging from 42703~86145 Hz, whereas the large phononic crystal exhibits a bandgap ranging from 50.2~101.4 Hz. Note that in the manuscript, wave localization is achieved at 77.0 Hz, which is almost in the middle of the range of 50.2 ~ 101.4 Hz. Hence, to generate the extremely low bandgap including 77.0 Hz, the general phononic crystal based on Bragg-scattering should have a length that is approximately 850 times larger.


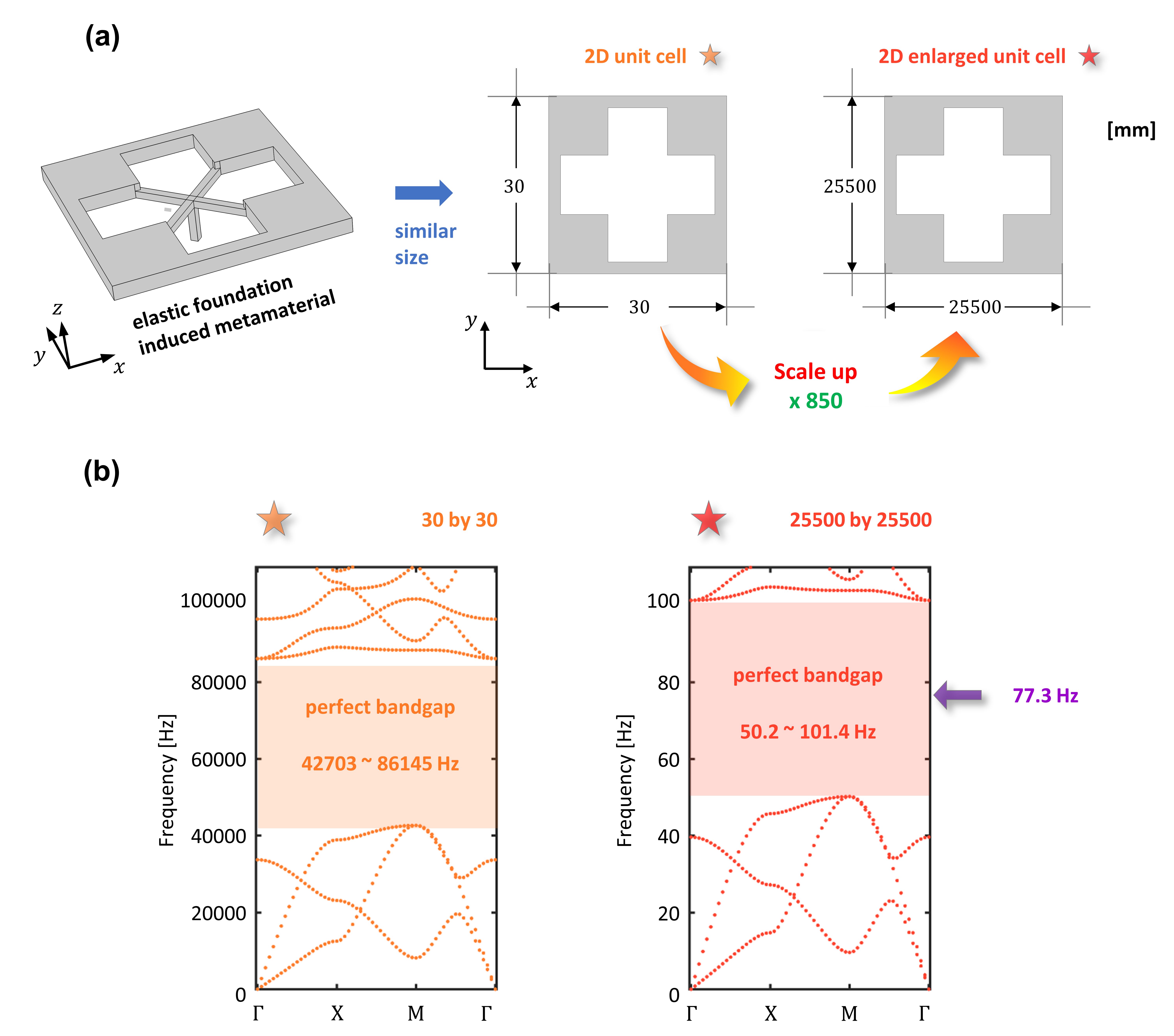


**Fig. S1.** Size comparison of unit cells. **(a)** Preparation of similar size and 850 times the unit cell, **(b)** dispersion curves with perfect bandgap.
